# Cognitive and Motivational Requirements for the Emergence of Cooperation in a Rat Social Game

**DOI:** 10.1371/journal.pone.0008483

**Published:** 2010-01-13

**Authors:** Duarte S. Viana, Isabel Gordo, Élio Sucena, Marta A. P. Moita

**Affiliations:** 1 Fundação Champalimaud Neuroscience Program at Instituto Gulbenkian de Ciência, Oeiras, Portugal; 2 Instituto Gulbenkian de Ciência, Oeiras, Portugal; 3 Departamento de Biologia Animal, Faculdade de Ciências da Universidade de Lisboa, Lisboa, Portugal; 4 Centro de Biologia do Desenvolvimento, Instituto Gulbenkian de Ciência, Oeiras, Portugal; Georgia State University, United States of America

## Abstract

**Background:**

Game theory and the Prisoner's Dilemma (PD) game in particular, which captures the paradox of cooperative interactions that lead to benefits but entail costs to the interacting individuals, have constituted a powerful tool in the study of the mechanisms of reciprocity. However, in non-human animals most tests of reciprocity in PD games have resulted in sustained defection strategie*s*. As a consequence, it has been suggested that under such stringent conditions as the PD game humans alone have evolved the necessary cognitive abilities to engage in reciprocity, namely, numerical discrimination, memory and control of temporal discounting.

**Methodology/Principal Findings:**

We use an iterated PD game to test rats (*Rattus norvegicus*) for the presence of such cognitive abilities by manipulating the strategy of the opponent, Tit-for-Tat and Pseudo-Random, or the relative size of the temptation to defect. We found that rats shape their behaviour according to the opponent's strategy and the relative outcome resulting from cooperative or defective moves. Finally, we show that the behaviour of rats is contingent upon their motivational state (hungry versus sated).

**Conclusions/Significance:**

Here we show that rats understand the payoff matrix of the PD game and the strategy of the opponent. Importantly, our findings reveal that rats possess the necessary cognitive capacities for reciprocity-based cooperation to emerge in the context of a prisoner's dilemma. Finally, the validation of the rat as a model to study reciprocity-based cooperation during the PD game opens new avenues of research in experimental neuroscience.

## Introduction

A central feature of the human species is its seemingly evolutionarily unprecedented capacity to establish cooperative interactions between non-related individuals. However, many examples describing similar behaviours in other animals revealed that this capacity is not exclusive to our species [1], questioning a simple Darwinian competition scenario for the evolution of cooperation. Over the last decades, several models have been put forward to solve this adaptive paradox such as kin selection, mutualism and reciprocity [2]–[5]. Nevertheless, when tested in natural populations and in laboratory conditions, some types of cooperation have been difficult to validate. In particular, evidence for reciprocity has not been free from controversy despite the abundance of reported cases including vampire bats [6], tree swallows [7], sticklebacks [8], impala [9], blue jays [10], cotton-top tamarin monkeys [11], red-winged blackbirds [12] and pied flycatchers [13].

Several mechanistic causes for the emergence of reciprocity-based cooperation, during the interaction between two individuals, have been put forward. One of these emphasizes pro-social propensity of the interacting individuals, in that a cooperative act constitutes a truly altruistic behaviour emerging from a reward value attributed to the perception of benefit to others [14]. Alternatively, from a strictly economic perspective, it is proposed that animals cooperate whenever it entails a benefit, either immediate or in the future, regardless of the consequence of its action to the other interacting individual [15]. These two apparently opposing views may both explain to some degree the emergence of cooperation. Indeed, cooperation in humans is sensitive to both pro-social and economic factors [16], [17].

Game Theory has proven to be instrumental in the study of social behaviour, as it formalizes mathematically the outcomes associated with the decisions of two or more interacting individuals, framing in economic terms the conditions for reciprocity [17], [18]. In the prisoner's dilemma (PD), the most studied game, cooperation leads to benefits but entails costs to the interacting individuals [19]–[21]. In the PD game, players can either cooperate or defect. If both cooperate they receive a higher payoff (Reward, R) than if they both defect (Punishment, P), but if one defects when the other cooperates, the defector receives the highest payoff (Temptation, T) whereas the cooperating individual receives the lowest (Sucker, S). The resulting payoff matrix follows the rule T>R>P>S. If the game is played only once the best strategy is defection, however cooperation can be stable if PD is played repeatedly (iterated PD). Several strategies have proven to lead to stable cooperation in the iPD game, of which Tit-for-Tat, the winning strategy in the now classical Axelrod's iPD tournament [18], [19], has been extremely successful. In this reciprocating strategy players start by cooperating and subsequently repeat the choice of the opponent on the previous game iteration.

The success of reciprocating strategies in theoretical models of iPD games has been corroborated experimentally by numerous reports on the emergence of reciprocal cooperation between human subjects while playing iPD games [22]–[24]. However, there are few reports of cooperation between non-human animals in an iPD game. It has been shown that corvids can cooperate in an iPD game if previously trained to cooperate under a mutualistic matrix, where mutual cooperation yields the highest payoff at no cost [10]. Furthermore, this maintenance of cooperation, after the switch to a prisoner's dilemma matrix (but see [25]), was only possible through the elimination of the effect of temporal discounting, i.e., the decrease in incentive value as the time to receive the future reward increases [21]. Importantly, this set of conditions that enabled cooperation in the iPD game constituted a significant deviation from the conditions in natural populations in which reciprocity might take place. First, it is unlikely that reciprocity between interacting animals is established after previous mutualistic interactions between the same individuals. Second, reciprocity is based on cooperative action that will be reciprocated by the other individual in a future interaction, this by definition involves temporal discounting. Indeed, in non-human animals, experimental evidence for the use of reciprocating strategies has fallen short of expectations as most tests have resulted in sustained defection strategies [26]–[28]. As a consequence, it has been suggested that non-human animals may have not evolved the necessary cognitive abilities to engage in reciprocity, namely numerical discrimination, memory and control of temporal discounting [21], [29].

Recently, it has been shown that rats can display generalized reciprocity [30] and the probability of a cooperative action is highest when it constitutes a reciprocating act toward a previously cooperative individual, direct reciprocity [31]. The remarkable finding that rats can be reciprocating animals, even though in the reported task there was no cost in cooperating nor temptation to defect, led us to re-visit the interactions between two rats during an iPD game where both cost and temptation are present.

## Results

### Emergence of Cooperation in an iPD Game under an Imposed TFT Strategy

**Figure 1:**
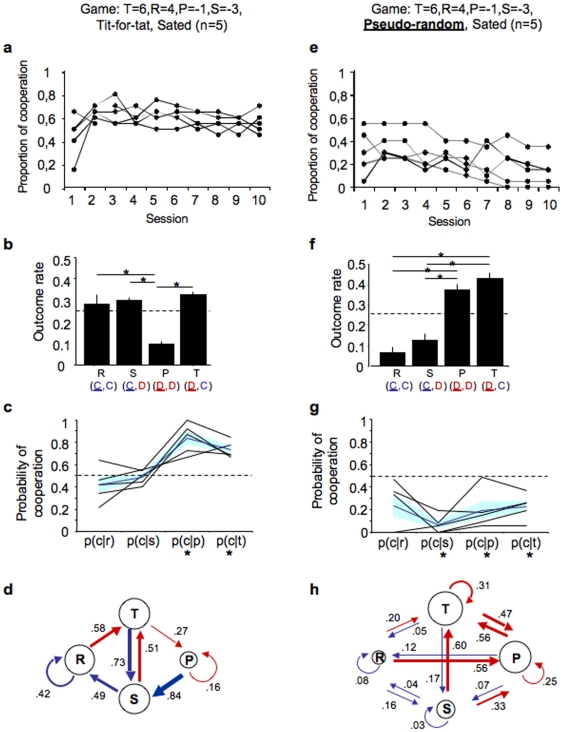
Rats adapt their behaviour to the strategy of the opponent. Sated rats playing under a matrix T = 6, R = 4, P = −1, S = −3 against a Tit-for-Tat (TFT) opponent (**a–d**) or a pseudo-random (PR) opponent (**e–h**). (**a**,**e**) Line graph showing the cooperation rate of each rat throughout the game sessions. (**b**,**f**) Bar graph shows incidence rate of all outcomes when cooperation reaches stability (mean±s.e.m.; see Materials and Methods). For each outcome, the moves of the target (underlined) and of the stooge rat are indicated in the graph legend. Asterisks denote significant difference between means (Multiple pairwise comparisons, using Nemenyi's Procedure, two tailed, Bonferroni corrected, *P*<0.0083). (**c**,**g**) Line graph showing probability of cooperation (at trial, t = 0) after x outcome (at trial, t = −1), P(C_0_|X_−1_), where X = T, R, P or S. Black lines represent P(C_0_|X_−1_), for each target rat, the dark blue line represents the mean P(C_0_|X_−1_) and the light blue band shows the s.e.m. (**d**,**h**) diagram showing the probability of transition between outcomes. Arrows represent transitions: driven by cooperation in blue, and driven by defection in red (arrow thickness proportional to transition probability). Asterisks denote significant difference from chance *P*<0.0083 (see Materials and Methods). T, temptation; R, reward; P, punishment; S, Sucker; C, cooperation; D, defection.

Our iPD game was played in a double T-maze in which choosing between arms was arbitrarily defined as cooperation (C) or defection (D). The payoff matrix was composed of both rewards and punishments, where T and R trials led to the delivery of food pellets and P and S trials led to the delivery of tail pinches (Table 1). After verifying that rats can discriminate between the different outcomes of our game matrix, we asked whether cooperation may emerge and be sustained during an iPD game when one of the players uses a reciprocating strategy, namely TFT. To this end, we fixed the strategy of one of the rats (the stooge), by placing it in the cooperation (C) or defection (D) compartment of the box (see Materials and Methods), as follows: on the first trial the stooge rat was placed in C, and for the remaining trials it was place in C or D according to the TFT rule. As expected, during the first game session, there was no significant cooperation. However, from the second session onwards, significant cooperation levels emerged (Figure 1a). The average rate of cooperation in the second session reached 0.63 (±0.01), and was maintained until the end of the game (average cooperation across sessions where behaviour was stable, 5 through 10, see methods, 0.58±0.01, n = 5). Strikingly, very similar cooperation rates have been found in human subjects when playing against a TFT opponent [22], [32].

**Table 1:** Prisoner's Dilemma Payoff Matrix.

	Stooge Cooperates	Stooge Defects
Target **C**ooperates	R = 4 pellets	S = 3 tail pinches
Target **D**efects	T = 6 pellets	P = 1 tail pinch

The table shows the choices of the two players (cooperate or defect) and the resulting payoffs (with the exception of experiments shown in Figure 3 and Figure S2 top panel). The payoffs shown result from cooperation or defection choices by the target rat (horizontal rows), when the stooge rat (columns) either cooperates or defects. R- reward, T- temptation, P- punishment, S- sucker.

In this game, the frequency of mutual cooperation was significantly higher than that of mutual defection (reward, R, and punishment, P, trials, respectively). However, a sizable incidence of temptation (T) and sucker (S) trials was observed (Friedman's ANOVA testing for effect of outcome, Q(3) = 53.485, two-tailed *P*<0.0001). Also, punishment was lower than all other outcomes (Figure 1b). This pattern results from the fact that after a defective move the probability of cooperating was high, and after a cooperative move the probability of cooperating was the same as that of defecting (probability of cooperating after defection: p(C_0_|P_−1_) = 0.84 and p(C_0_|T_−1_) = 0.73; probability of cooperating after cooperation: p(C_0_|R_−1_) = 0.42 and p(C_0_|S_−1_) = 0.49, Figure 1c). That is, the probability of repeating a defection move was very low, leading to infrequent punishment outcomes. In contrast, the probability of repeating a cooperative move was not different from chance. That is, after cooperating in a given trial, staying in mutual cooperation or defecting to obtain the temptation payoff in the next trial was equally likely. In summary, rats cooperated more often than they defected. Once rats defected they quickly reverted to cooperation, avoiding being stuck in mutual defection cycles (Figure 1d). In addition, after an initial learning period, the strategy adopted by the different rats was remarkably similar (see Figure 1c and Figure S1 for individual performances). In conclusion, rats when playing against a reciprocating opponent will display a behaviour that is composed of both mutual cooperation (reward trials) and alternating reciprocity (alternating temptation and sucker trials).

### Rats Adapt Their Behaviour to the Strategy of the Opponent

If the emergence of cooperation between two interacting individuals is contingent upon the adoption of a reciprocating behaviour by at least one of them, then, if the stooge rat uses a non-reciprocating strategy, cooperation rates should decrease. To test this hypothesis we fixed the stooge rat in a pseudo-random strategy, so that the choices of the target rat would not influence subsequent moves of the stooge. During any given session the pseudo-random stooge rat cooperates on average 50% of the trials. The sequence of defective and cooperative moves was randomized, however, no more that 3 consecutive defective or cooperative moves were allowed.

When playing against a random opponent, as in a single-shot game, the best strategy is to defect. Indeed, we found that against a pseudo-random strategy, the rat will predominantly defect (average cooperation rate across sessions 5 to 10, 0.20±0.02, n = 5). Cooperation in this game was low irrespective of the outcome of the previous trial (Figure 1e and f), resulting in low rates of both reward and sucker trials, and high incidence of temptation and punishment trials (Friedman's ANOVA testing for effect of outcome, Q(3) = 77.915, two-tailed *P*<0.0001.Figure 1g). This observation is a reflection of the global defection strategy adopted by these animals (see Figure S2 for individual performances). Furthermore, these data reinforce the conclusions from the previous experiment (Figure 1a–d), in that they show that rats can cooperate in an iPD game and that their behaviour depends on the strategy adopted by the opponent. Thus, in our set-up, reciprocation is necessary for the emergence and sustainability of cooperation.

Results from these experiments (TFT and PR games) show that rats display consistent differences in their behaviour depending on the opponent's strategy. Nonetheless, in both cases rats are suboptimal, i.e. the behaviour they adopt does not yield the best possible outcome. If optimal, when playing against TFT, rats should always cooperate [18], [19], which would yield the highest number of rewards and no punishments (observed cooperation rate was ∼60%). In contrast, when Playing against PR, rats should always defect [18], [19], again maximizing reward and minimizing punishment (observed defection rate was ∼80%). A common observation in two alternative choice paradigms is that animals do not adopt an optimal strategy, following, instead, strategies such as the matching rule [33]. Matching behaviour is typically observed when animals face choices associated with different reward probabilities, in which case animals match their choice rate to the reward rate at each choice (e.g., they chose 80% of the time the action that has 0.8 probability of being rewarded) [34]. Nevertheless, the behaviour of rats in the iPD game cannot be explained by a simple matching rule [34], in which the probability of cooperating or defecting depends on the relative magnitude of the payoff obtained from either choice. Indeed, we found that the only instance in which the choice between cooperation and defection conforms to the matching rule corresponds to the choice between positive payoffs (i.e. after a reward trial, R, or a sucker trial, S) during the TFT game (Figure 1a–d). This rule does not apply either for choices between negative outcomes (i.e. after a temptation trial, T, or a punishment trial, P), nor to the game where the stooge is playing a pseudo-random strategy, where on each trial the rat's choice may lead to one of four possible outcomes, both positive and negative (see Figure S3).

**Figure 2:**
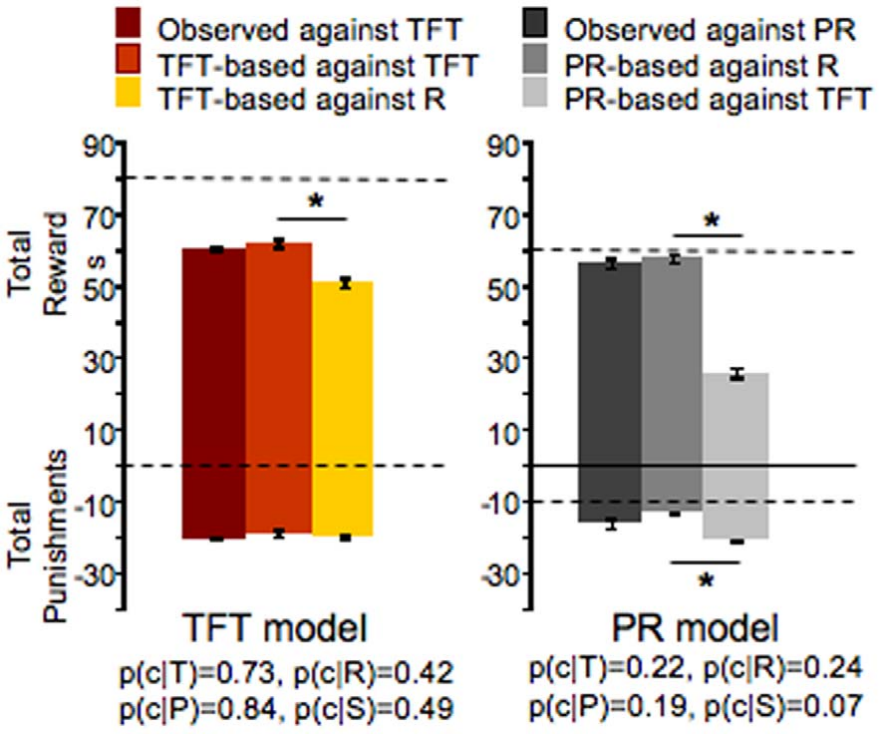
Rats adjust their strategy to the behaviour of the opponent. Simulated games using TFT and PR were performed using the empirically determined cooperation probabilities after each outcome, T, R, P and S, of the game (see Figure 1d and h). (**left panel**) Bar graph showing average outcome, rewards and punishments, per session (sum of 20 trials) of the experimental TFT game and, TFT-based simulation against TFT and Random. (**right panel**) Bar graph showing average outcome, rewards and punishments, per session (sum of 20 trials) of the experimental PR game and, PR-based simulation against TFT and Random. Dashed lines represent the theoretically predicted optimal values for a 20-trial session when the opponent is playing TFT (panel a) and Random (panel b).

Although the observed behaviour is suboptimal, and does not seem to conform to the matching rule it is clearly sensitive to the opponent's strategy, thus raising the question of whether the difference in the behaviour adopted by the rats is truly adjusted to the opponents' strategy. This question can only be addressed through game simulations where the behaviour adopted by the rats (observed behaviour) can be played out against different strategies, e.g. TFT and pseudo-random. To this end, we used the observed strategies of rats playing against TFT or PR (Figures 2d and 2h, respectively), to model games where these same strategies (which we call TFT-based and PR-based) were played against pure TFT or Random opponents. Next, we compared the outcome, in rewards and punishments, of the real games (observed behaviours in the TFT and PR games), with the game between TFT-based or PR-based (simulation base on observed behaviour) playing against pure TFT or random opponents. We found no difference between simulated TFT-based against TFT and the real TFT game, validating our simulation (unpaired two-tailed t-tests, Bonferroni corrected, showed, p>0.01) and that the simulated TFT-based rats did better when playing against a TFT than against a Random opponent (unpaired two-tailed t-tests, Bonferroni corrected, show significant difference for rewards p<0.0001, and no difference for punishments, p>0.01, Figure 2). Conversely, the simulated PR-based rats did better when playing against a Random than against a TFT opponent (unpaired two-tailed t-tests, Bonferroni corrected, for rewards p<0.0001 and for punishments p<0.01), whereas no difference between the simulated and the real game was found (p>0.01, Figure 2). Thus, although suboptimal, the strategy adopted by rats in our experiments was adjusted to the opponent's behaviour. Furthermore, we found that rats were closer to optimality when playing against PR than against TFT. This result may be explained by the fact that under a PR strategy the game is equivalent to successive single-shot games, whereas under TFT it is equivalent to an iterated game, where the outcome of a game iteration depends on the previous one. Therefore, playing the PD game under a PR strategy may pose a simpler problem to the players.

### Rats Adapt Their Behaviour to the Economic Terms of the Payoff Matrix

**Figure 3:**
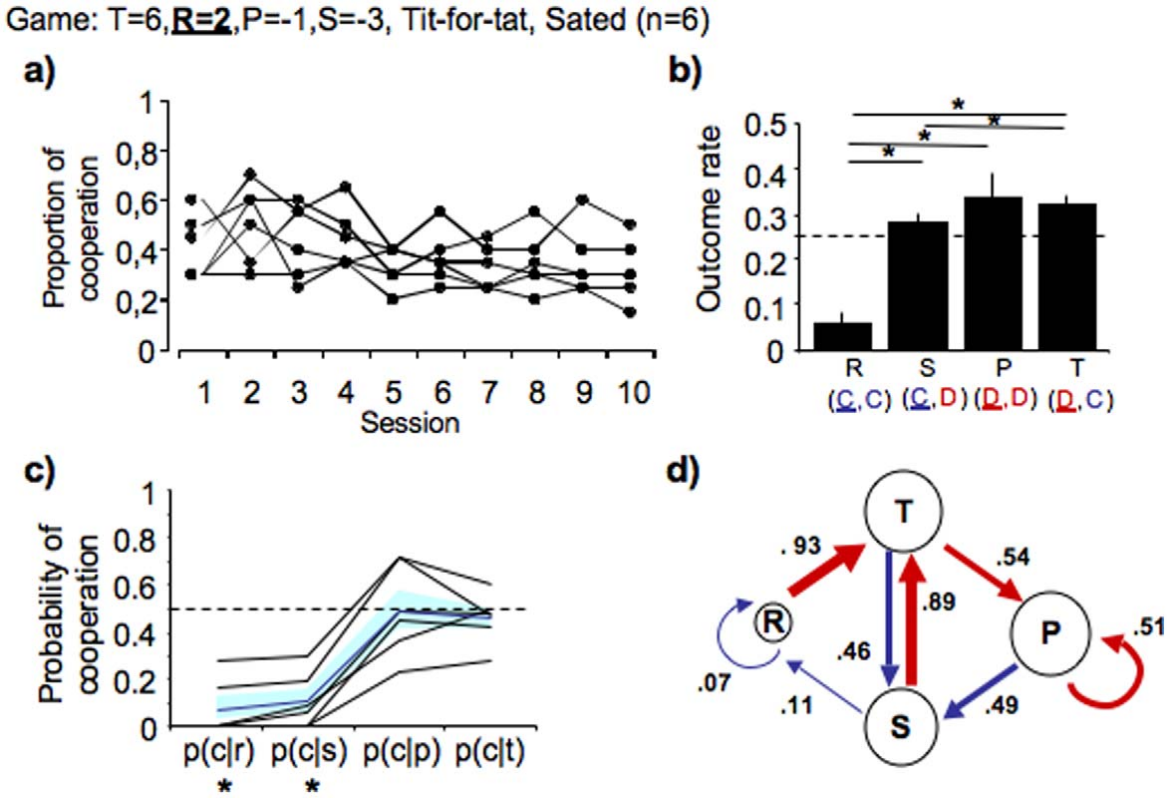
Rats shape their strategy according to economic terms of the game. (**a**) Line graph showing the cooperation rate of each rat throughout the game sessions. (**b**) Bar graphs show incidence rate of all outcomes when cooperation reaches stability. (**c**) Line graph showing probability of cooperation after each outcome (**d**) Diagram showing the probability of transition between outcomes. All graphs and diagrams and notations as in Figure 1. Asterisks denote significant difference from chance *P*<0.0083 (see Materials and Methods).

In an iPD game, the highest immediate payoff results from a temptation trial. However, the highest gain along all sessions is achieved when both players always cooperate (resulting in 4 food pellets every trial). Thus, when playing against a TFT opponent and in full knowledge of the opponent's current move the target player should always cooperate. However, if the temptation to defect is high, subjects will eventually defect. As previously seen, when playing against TFT, rats in our iPD game cooperate more often than they defect, but show high incidence of temptation trials which are immediately followed by cooperation. One possibility is that rats adopted a mixed strategy (mutual cooperation and alternating reciprocity) because, even though the temptation to defect is significant, mutual cooperation entails a higher payoff than pure alternating reciprocity. If this is true, then decreasing the outcome of a reward trial (from 4 to 2 food pellets) while maintaining the level of temptation (6 pellets), should maintain the levels of alternation between T and S trials, while decrease the levels of mutual cooperation (R trials). As predicted, we found that rats showed similar T and S levels as in the first experiment (where R = 4 and T = 6), whereas the incidence of mutual cooperation was significantly lower than that of mutual defection (Friedman's ANOVA testing for effect of outcome, Q(3) = 66.630, two-tailed *P*<0.0001, n = 6. See Figure 3b and Figure S2 for individual performances). Resulting from a low incidence of mutual cooperation the cooperation rate in this game as low (average cooperation rate across sessions 5 to 10, 0.34±0.02, n = 6) (Figure 3a). This shows that the economic terms of the iPD game are perceived by the rat and shape its adopted strategy indicating that rats are capable of numerical discrimination. In addition, these data suggest that rats are capable of discriminating between matrices where 2R>T+S (Figure 1) versus 2R<T+S (Figure 3). Furthermore, together with the previous experiments (see above), these data strongly suggest that rats remember the past history of the game, up to at least one trial back, since cooperation only emerged in the following conditions: 1) the opponent played TFT (which relies on the moves of the previous trial); 2) when the payoff for mutual cooperation was higher than alternating reciprocity, which only impacts payoffs accumulated over two or more trials. This result strengthens the previous finding that rats are capable of direct reciprocity which requires memory of previous interactions [29]. Altogether, our results show that rats have the cognitive capacity to engage in a reciprocating strategy.

### Rats' Behaviour Is Contingent upon Their Motivational State

**Figure 4:**
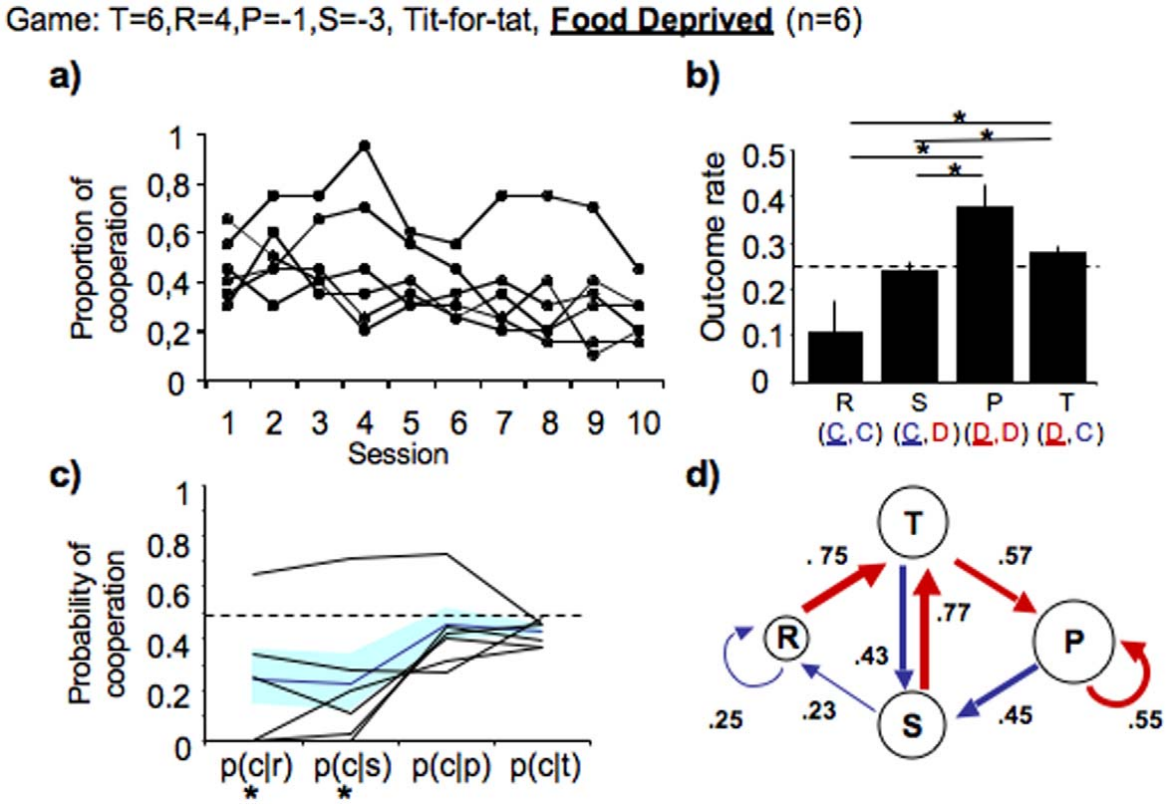
Rats shape their strategy according to their motivational state. (**a**) Line graph showing the cooperation rate of each rat throughout the game sessions. (**b**) Bar graphs show incidence rate of all outcomes when cooperation reaches stability. (**c**) Line graph showing probability of cooperation after each outcome (**d**) Diagram showing the probability of transition between outcomes. All graphs and diagrams and notations as in Figure 1. Asterisks denote significant difference from chance *P*<0.0083 (see Materials and Methods).

Several explanations for the lack of cooperation in previous studies have been put forward such as high impulsiveness of non-human animals [35]. Indeed, it has been demonstrated experimentally that impulsiveness and temporal-discounting do play a role in the animals' cooperation rate [21], [35], [36]. Importantly, in all these studies animals were moderately food deprived. In rats, food deprivation can alter impulsiveness and incentive value of food rewards [37]. In our experiments rats had free access to food, and thus were sated, which could explain our success in observing cooperation contrasting with previous findings. To test this possibility we changed the motivational state of the rats, through moderate food deprivation. As predicted, when food deprived, rats playing an iPD game against TFT failed to sustain high levels of cooperation (average cooperation rate across sessions 5 to 10, 0.35±0.03, n = 6) (Figure 4a and c). Indeed the incidence of cooperative trials, R and S, was lower than that of defective trials (Friedman's ANOVA testing for effect of outcome, Q(3) = 43.135, two-tailed *P*<0.0001, Figure 4b and Figure S2 for individual performances). This highlights the central role for motivational state in decision-making and its consequences in the behavioural outcome of two interacting individuals.

## Discussion

**Figure 5:**
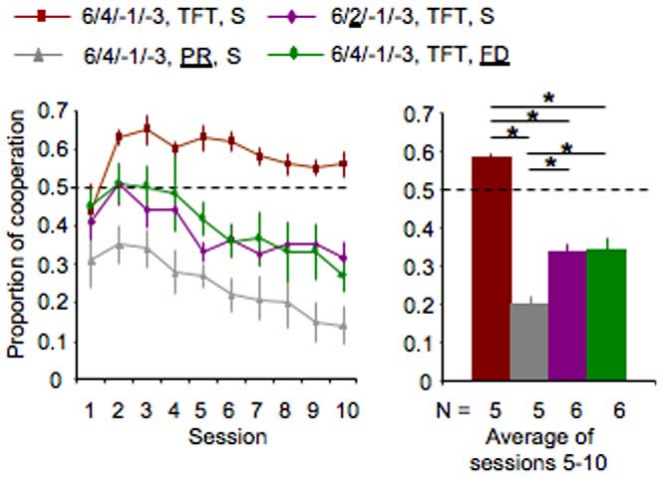
Cooperation levels vary with the different iPD games. Left panel shows time-course of cooperation rate along the ten game sessions for each iPD game (mean±s.e.m.). Each line represents one of the games tested. Right panel shows proportion of cooperation in each iPD game (mean±s.e.m.) when cooperation reaches stability (see methods). Each bar represents one game (same colour as in line graph). Asterisk, denote significant difference between means (Multiple pairwise comparisons, using Dunn's Procedure, two tailed Bonferroni corrected, *P*<0.0083).

We show that for rats playing an iPD game, the cooperation rate is modulated by the strategy of the opponent; the relative size of the reward resulting from cooperation and defection; and the motivational state of the animals (Kruskal-Wallis testing for effect of game, K = 85.452, *P*<0.0001, Figure 5). Taken together these results show that rats possess cognitive capacities necessary for the control of impulsivity, numerical discrimination and memory, compatible with the adoption of behaviours, including cooperation, that conform to game theory predictions. Furthermore, we show that engaging in cooperation can be modulated by the motivational state of the animal revealing that environmental factors may impinge on the perception of the strict economic outcome of social interactions. In addition, this behaviour may be modulated by social interactions, as it is possible that the rats were using the opponent as a cue. Indeed, it has been shown that humans modulate their propensity to cooperate depending on the identity of the opponent (e.g, computer *vs* other human) [24]. Also, in our iPD game choices were sequential raising the question of whether cooperation would have emerged and be maintained in a simultaneous choice game. Further work is needed to specifically address the importance of such factors in establishing reciprocity-based interactions between rats.

In conclusion, our results reinforce the notion that rats are capable of complex computations [38]–[40] and imply that the evolutionary origin of the cognitive basis for reciprocity is rooted deeply in the phylogeny of mammals. Finally, our findings may widen the scope for future studies of decision-making mechanisms in the context of social interactions [41], [42] using the rat model system.

## Materials and Methods

### 1. Ethics Statement

The Instituto Gulbenkian de Ciência follows the Portuguese Guidelines, which comply with the European Directive 86/609/EEC of the European Council.

### 2. Subjects

The experiments were performed using male non-litter mates of the outbred Sprague Dawley rat strain, from Charles River, Barcelona, Spain. All animals were housed in pairs under 12 h light/dark cycle. Experiments were conducted during the light period. Before starting the experiment, all rats were habituated for one week to the experimenter and to the novel food used for the positive reinforcements in the iPD game. Each experiment used naive rats and all rats within a game played against the same stooge. In each game 5 to 6 target rats were used (see corresponding Figures for sample size). For the experiments using sated rats, subjects had free access to food and water, whereas, for the experiments using food-deprived rats, animals had restricted access to food and kept at 85% of their *ad libitum* weight.

### 3. Apparatus

**Figure 6:**
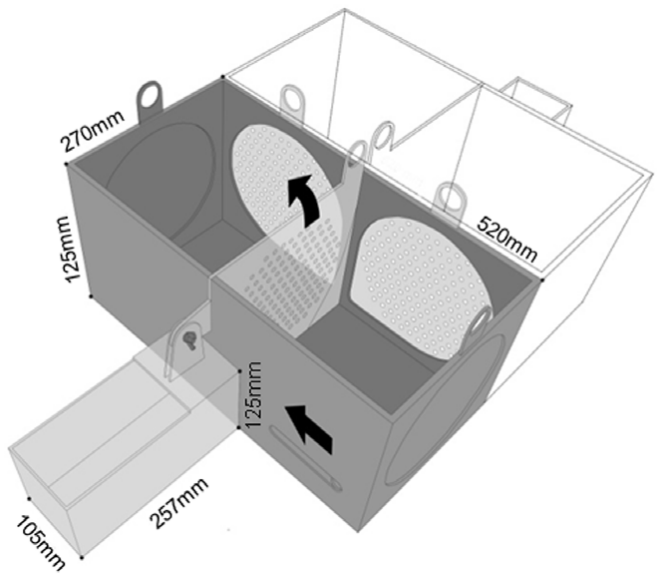
T-maze diagram. Diagram of the double T-maze used in the presented experiments. One T-maze is represented in grey with its respective start box and two choice compartments. In dashed lines is shown the identical opposing T-maze. Arrows show the movement direction of the start box door and of the partition between compartments.

The apparatus consists of a double T-maze made of plexiglass (Figure 6). Each T-maze consisted of a small start box that gave access, through a sliding door, to two compartments (Figure 6). The two compartments of each T-maze were separated by a movable partitioning wall. The sidewalls of each T-maze were black. The walls adjacent to the opposing T-Maze, and the partitioning wall between compartments within the maze were transparent and perforated, so that rats could see and smell each other from all locations in the maze.

### 4. Payoff Matrix

We used a Prisoner's Dilemma game matrix, in which T>R>P>S. Preliminary evidence showed that, as predicted by Stevens and Clements [43], heterogeneous matrices (with both positive, R and T, and negative, P and S, outcomes) lead to higher cooperation rates as compared to homogeneous, all-reward, matrices (data not shown). Therefore, we used a payoff matrix composed of both rewards and punishments, where temptation and reward trials led to the delivery of food pellets (Bio-Serv 45 mg precision food pellets) and punishment and sucker trials led to the delivery of tail pinches (forceps were used to deliver tail pinches close to the tip of the rats' tail). Table 1 shows the payoff matrix used in our game.

We first verified that rats can discriminate the difference between temptation and reward outcomes (6 vs. 4 food pellets respectively), and between punishment and sucker outcomes (1 vs. 3 tail pinches respectively). In these experiments only one T-Maze was used and simple preference tests were performed. Rats were placed in the start box and given a choice between the two compartments of the T-maze. For the positive reinforcement test, 6 pellets (T outcome) were delivered in one compartment and 4 pellets (R outcome) in the other (high and low rewards were delivered in a counterbalanced fashion in the left and right compartment). We found that over 5 days with one session of 20 trials, preference for the 6-pellet compartment steadily increased reaching 84±2% by the last day. G-test shows that choice of high reward was significantly different from chance, G_P_ = 61.50, P = 4.4×10^−15^ (see Materials and Methods, section 6.3). For the negative reinforcement test a similar experiment was performed, where 1 tail pinch was delivered in one compartment and 3 tail pinches in the other. A preference for the 1 tail pinch compartment emerged in the second half of the first session and remained stable around 60% for the remaining training sessions (on the last session average choice of 1 tail pinch was 59±3%, significantly different from chance, G_P_ = 4.06, P = 0.04). These results show that rats could discriminate 6 over 4 food pellets, and 1 over 3 tail pinches.

### 5. iPD Game

All iPD games were played for 10 consecutive days consisting of one daily session, of 20 trials each. The two compartments of each T-Maze were arbitrarily defined as cooperation (C) or defection (D) compartments (counterbalanced across rats). Thus, in our iPD game a cooperating or defecting act was defined as entering the C or D compartment respectively. For consecutive target rats cooperation or defection was ascribed to opposing compartments. This experimental design guarantees that odour cues from the previous target rat would elicit the opposite response (cooperate or defect) from the following target rat. For each game one of the rats, the stooge, was assigned to play a fixed strategy, either Tit-For-Tat or Pseudo-Random, and the other rat (the target) could freely choose between C and D. A new stooge was used for each game but it was the same for all rats within a game. On each trial of the game, the stooge was placed in C or D (according to the *a priori* defined strategy), after which the target rat was placed in the start box of the adjacent T-maze and was allowed to go for D or C (free choice). Given that the partition wall between mazes was transparent and perforated, the target rat can use the placement of the stooge as a cue to guide its choice. Once the decision was made, i.e. when all four paws were inside one of the compartments, the experimenter closed the partition and delivered the reinforcement, according to the payoff matrix of the game. If the target rat did not choose a compartment within 30 seconds of the beginning of the trial, the experimenter slowly closed the partition prompting the rats to choose one of the two compartments. The target rat was then removed from the T-maze and a new trial started with next target rat. For each target rat the average inter-trial interval was 4–5 minutes (corresponding to the amount of time it took to run all rats, typically 4 were run on any given session). Before the first session of the game, rats were exposed to the T-maze (habituation phase), three days without the stooge in the adjacent T-maze and five days with the stooge in the adjacent T-maze (5 min/day).

### 6. Statistical Analysis

All statistical analysis, except for the G-tests (which were calculated manually using Excel from Microsoft Office, see paragraph III), where performed using XSTAT, from Microsoft. Since the data analyzed did not follow normal distributions (as shown by Shapiro-Wilk normality tests) non-parametric statistical tests were used for analysis II, III and IV.

#### 6.1–Stability of cooperation

In order to analyse the strategy adopted by the rats in the different games, we pooled the data from the sessions in which cooperation rates were stable. To identify when the cooperation rate stabilized, for each game we plotted the cooperation rate for all rats across sessions (note that no animal was excluded from the analysis). Next we fitted several linear models to the data, where the first model included all sessions, and in the successive models the data included would slide by one session (model 1 included sessions 1 through 10; model 2 included sessions 2 through 10 and so on). We found that for all games from session 5 onwards the slope of the linear fit was not different from zero. Thus, for all analysis of the rats' performance the data was pooled from sessions 5 through 10.

#### 6.2–Rate of incidence of the different outcomes, T, R, P and S

To compare mean rate of the different outcomes, for each game we first performed a Friedman's ANOVA (with outcome as single within subject factor). When significant, α = 0.05, multiple post-hoc pairwise comparisons using the Nemenyi's procedure/Two-tailed tests were performed, with the Bonferroni corrected α value of 0.0083.

#### 6.3–Probability of cooperation after each outcome

To test whether the probability of cooperation after each outcome was different from chance, i.e. 0.5, we performed G-tests. We calculated the parameter G_P_ to test for deviations from the theoretical distribution [44]. Bonferroni corrected significance value corresponds to α = 0.01.

#### 6.4–Cooperation rate in the different games

To compare the mean cooperation rate observed in the different games a Kruskal-Wallis ANOVA was performed using game as a single between-subject factor. Multiple post-hoc pairwise comparisons using the Dunn's procedure/Two-tailed test were performed, with the Bonferroni corrected α value of 0.0083.

### 7. Simulation of Games Based on the Observed Behaviour

In order to assess whether the behaviour adopted by rats when playing against TFT (observed behaviour) would yield a worse outcome if the same strategy would be adopted against a Random opponent, we simulated games where the observed strategy was played against TFT (modelling the real game) or against a random opponent. To model the observed behaviour we used the probability of cooperation after each of the game's outcomes (T,R,P,S), averaged across the five rats that played TFT (P(C_0_|T_−1_) = 0.73, P(C_0_|R_−1_) = 0.42 P(C_0_|P_−1_) = 0.84, P(C_0_|S_−1_) = 0.49). This model (consisting of the above cooperation probabilities) corresponds to the simulated TFT-based player. Using this model we simulated a game where the opponent was playing either pure TFT or pure Random. The simulation was run 5 times for each opponent. First, to validate our model, the average outcome for the simulated game against TFT was compared to the average outcome obtained by rats playing the real game (observed outcome). Next, the outcome of the simulated game against TFT was compared to that against Random.

The same procedure was used to model the observed behaviour when rats played against a pseudo-random stooge rat, so that we could assess whether the behaviour adopted by rats when playing against PR (observed behaviour) would yield a worse outcome if the same strategy would be adopted against a TFT opponent. For the simulated PR-based player we used the following probabilities: p(C_0_|T_−1_) = 0.22, p(C_0_|R_−1_) = 0.24 p(C_0_|P_−1_) = 0.19, p(C_0_|S_−1_) = 0.07 (average probabilities across the five rats that played against PR). Note that in the real game the stooge rat was playing a pseudo-random strategy, in such way that there was never more than 4 times the same move (C or D), whereas the virtual rat played a pure random strategy.

The simulations were run in Excel from Microsoft Office. Comparisons of the outcome from the different simulated games unpaired, two-tailed T-tests were performed, with the Bonferroni corrected α value of 0.01.

## Supporting Information

Figure S1 Diagram showing the probability of transition between outcomes of individual rats. Arrows represent transitions: driven by cooperation in blue, and driven by defection in red (arrow thickness proportional to transition probability). In all panels: T, temptation; R, reward; P, punishment; S, Sucker; C, cooperation; D, defection.
(0.13 MB TIF)

Figure S2 Diagram showing the probability of transition between outcomes of individual rats. Subjects shape their strategy according to the iPD game conditions (each game differs from game 1 for the highlighted condition). Arrows represent transitions: driven by cooperation in blue, and driven by defection in red (arrow thickness proportional to transition probability). In all panels: T, temptation; R, reward; P, punishment; S, Sucker; C, cooperation; D, defection.
(0.12 MB TIF)

Figure S3 Comparison between observed behaviour and matching behaviour. The figure shows the observed probability of cooperation after each outcome, Reward, Sucker, Punishment and Temptation, black bars (mean±s.e.m), together with the expected probability of cooperation if rats would be matching for reward (p(C0|R-1) and p(C0|S-1)) or punishment (p(C0|P-1) and p(C0|T-1)) magnitudes, white bars. The observed behaviour approached that of matching only for the game in a), when rats were choosing between 6 or 4 food pellets, i.e., after a reward or sucker trial. Note that this analysis is not possible for the game where rats were playing against a pseudo-random stooge, because all transitions between outcomes were possible, and thus, rats had to choose between rewards or punishments of different magnitudes, but also between rewards and punishment (in this case outcomes are not comparable, therefore matching does not apply).
(0.08 MB TIF)

## References

[1] Dugatkin LA (1997). Cooperation Among Animals: An Evolutionary Perspective..

[2] Pusey AE, Packer C, Krebs JR, Davies NB (1997). The ecology of relationships.. Behavioural Ecology - an Evolutionary Approach.

[3] Dugatkin LA (2002). Animal cooperation among unrelated individuals.. Naturwissenschaften.

[4] Nowak MA (2006). Five rules for the evolution of cooperation.. Science.

[5] West SA, Griffin AS, Gardner A (2007). Social semantics: altruism, cooperation, mutualism, strong reciprocity and group selection.. Journal of Evolutionary Biology.

[6] Wilkinson GS (1984). Reciprocal food sharing in the vampire bat.. Nature.

[7] Lombardo MP (1985). Mutual restraint in tree swallows: a test of the Ti-For-Tat model of reciprocity.. Science.

[8] Milinski M (1987). Tit-For-Tat in sticklebacks and the evolution of cooperation.. Nature.

[9] Hart BL, Hart LA (1992). Reciprocal allogrooming in impala, Aepyceros melampus.. Anim Behav.

[10] Stephens DW, McLinn CM, Stevens JR (2002). Discounting and reciprocity in an iterated prisoner's dilemma.. Science.

[11] Hauser MD, Chen KM, Chen F, Chuang E (2003). Give onto others: genetically unrelated cotton-top tamarin monkeys preferentially give food to those who altruistically give food back.. Proc R Soc London Ser B.

[12] Olendorf R, Getty T, Scribner K (2004). Cooperative nest defence in red-winged blackbirds: reciprocal altruism, kinship or by-product mutualism?. Proc R Soc London B.

[13] Krams I, Krama T, Igaune K, Mänd R (2008). Experimental evidence of reciprocal altruism in the pied flycatcher.. Behav Ecol Sociobiol.

[14] Camerer CF, Fehr E (2006). When does “economic man” dominate social behaviour?. Science.

[15] Trivers RL (1971). The evolution of reciprocal altruism.. The Quarterly Review of Biology.

[16] Fehr E, Fischbacher U, Gachter S (2002). Strong reciprocity, human cooperation, and the enforcement of social norms.. Human Nature.

[17] Sheldon KM (1999). Learning the lessons of tit-for-tat: Even competitors can get the message.. Journal of Personality and Social Psychology.

[18] Dugatkin LA, Dugatkin LA, Reeve HK (1998). Game theory and cooperation.. Game Theory and Animal Behaviour.

[19] Axelrod R (1984). The Evolution of Cooperation..

[20] Axelrod R, Hamilton WD (1981). The Evolution of Cooperation.. Science.

[21] Stephens DW, McLinn CM, Stevens JR (2002). Discounting and reciprocity in an iterated prisoner's dilemma.. Science.

[22] Harris AC, Madden GJ (2002). Delay discounting and performance on the prisoner's dilemma game.. Psych Rec.

[23] Wedekind C, Milinski M (1996). Human cooperation in the simultaneous and the alternating prisoner's dilemma: Pavlov versus generous Tit-For-Tat.. Proc Natl Acad Sci U S A.

[24] Rilling JK, Gutman DA, Zeh TR, Pagnoni G, Berns GS (2002). A neural basis for social cooperation.. Neuron.

[25] Kéfi S, Bonnet O, Danchin E (2007). Accumulated gain in a Prisoner's Dilemma: which game is carried out by the players?. Anim Behav.

[26] Flood M, Lendenmann K, Rappoport A (1983). 2#2 Games played by rats: different delays of reinforcement as payoffs.. Behav Sci.

[27] Clements KC, Stephens DW (1995). Testing models of non-kin cooperation: mutualism and the prisoner's dilemma.. Anim Behav.

[28] Hall SS (2003). Transitions between cooperative and non-cooperative responding in the pigeon's dilemma.. Behav Proc.

[29] Stevens JR, Hauser MD (2004). Why be nice? Psychological constraints on the evolution of cooperation.. TIGS.

[30] Rutte C, Taborsky M (2007). Generalized reciprocity in rats.. PLoS Biology.

[31] Rutte C, Taborsky M (2008). The influence of social experience on cooperative behaviour of rats (Rattus norvegicus): direct vs generalized reciprocity.. Behav Ecol Sociobiol.

[32] Yi R, Johnson MW, Bickel WK (2005). Relationship between cooperation in an iterated prisoner's dilemma game and the discounting of hypothetical outcomes.. Learn Behav.

[33] Williams BA, Mackintosh NJ (1994). Reinforcement and choice.. Animal learning and cognition.

[34] Herrnstein RJ (1961). Relative and absolute strength of responses as a function of frequency of reinforcement.. J Exp Anal Behav.

[35] Stephens DW, McLinn CM, Stevens JR (2006). Effects of temporal clumping and payoff accumulation on impulsiveness and cooperation.. Behav Proc.

[36] Baker F, Rachlin H (2002). Self-control by pigeons in the prisoner's dilemma.. Psych Bull Rev.

[37] Uslaner JM, Robinson TE (2006). Subthalamic nucleus lesions increase impulsive action and decrease impulsive choice - mediation by enhanced incentive motivation?. Eur J Neurosci.

[38] Fortin NJ, Wright SP, Eichenbaum H (2004). Recollection-like memory retrieval in rats is dependent on the hippocampus.. Nature.

[39] Kepecs A, Ushida N, Zariwala HA, Mainen ZF (2008). Neural correlates, computation and behavioural impact of decision confidence.. Nature.

[40] Murphy RA, Mondragón E, Murphy VA (2008). Rule learning in rats.. Science.

[41] Lee D (2008). Game theory and neural basis of social decision making.. Nat Neurosci.

[42] Watson KK, Platt ML (2008). Neuroethology of reward and decision making.. Philos Trans R Soc Lond B Biol Sci.

[43] Stephens DW, Clements KC (1998). Game theory and Animal Behaviour..

[44] Sokal RR, Rohlf FJ (1969). Biometry.

