# Supplementary figures and images for: Cognitive and Motivational Requirements for the Emergence of Cooperation in a Rat Social Game
Source: PLoS One. 2010 Jan 13;5(1):e8483. doi: 10.1371/journal.pone.0008483 (PMC2799661; doi:10.1371/journal.pone.0008483)

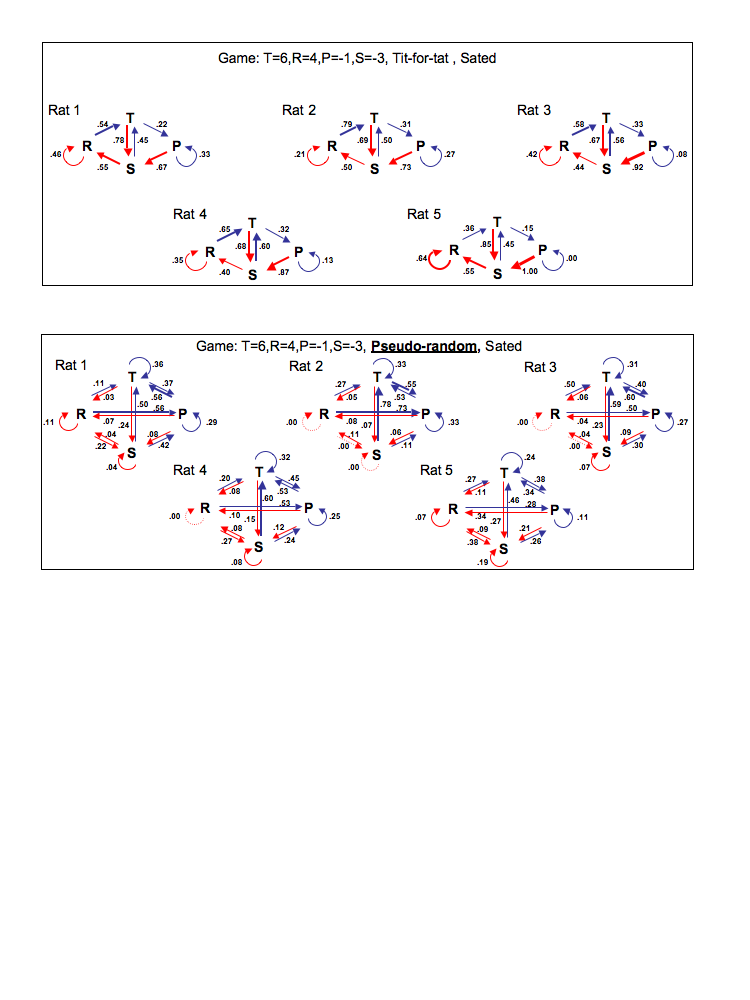

Supplement: Figure S1 — Diagram showing the probability of transition between outcomes of individual rats. Arrows represent transitions: driven by cooperation in blue, and driven by defection in red (arrow thickness proportional to transition probability). In all panels: T, temptation; R, reward; P, punishment; S, Sucker; C, cooperation; D, defection. (0.13 MB TIF) [file pone.0008483.s001.tif]

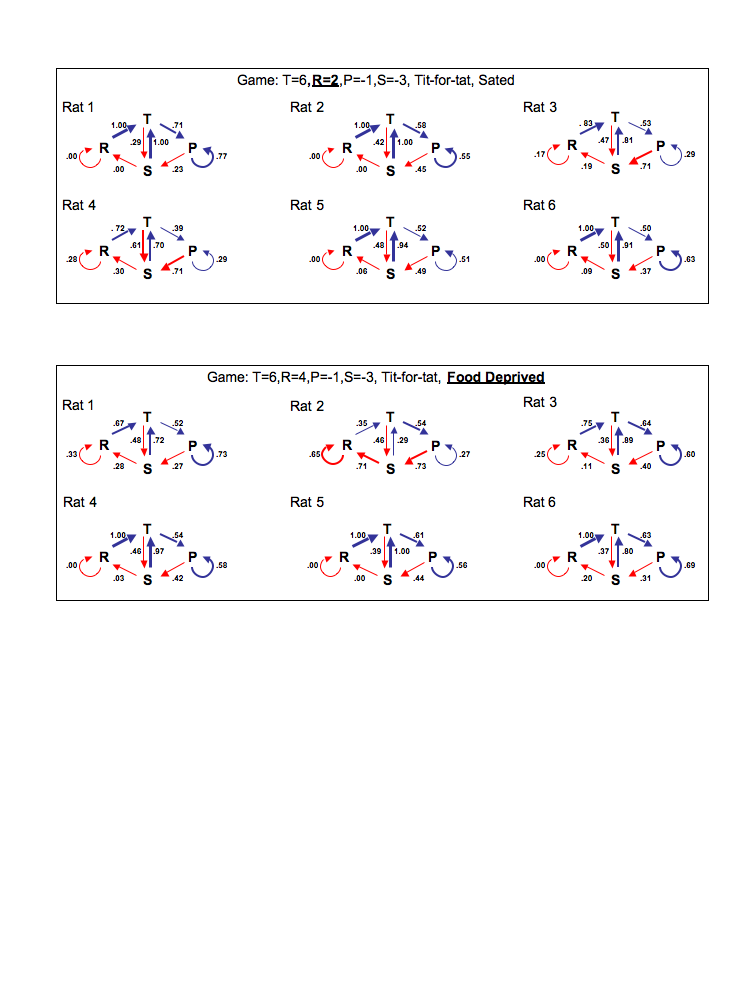

Supplement: Figure S2 — Diagram showing the probability of transition between outcomes of individual rats. Subjects shape their strategy according to the iPD game conditions (each game differs from game 1 for the highlighted condition). Arrows represent transitions: driven by cooperation in blue, and driven by defection in red (arrow thickness proportional to transition probability). In all panels: T, temptation; R, reward; P, punishment; S, Sucker; C, cooperation; D, defection. (0.12 MB TIF) [file pone.0008483.s002.tif]

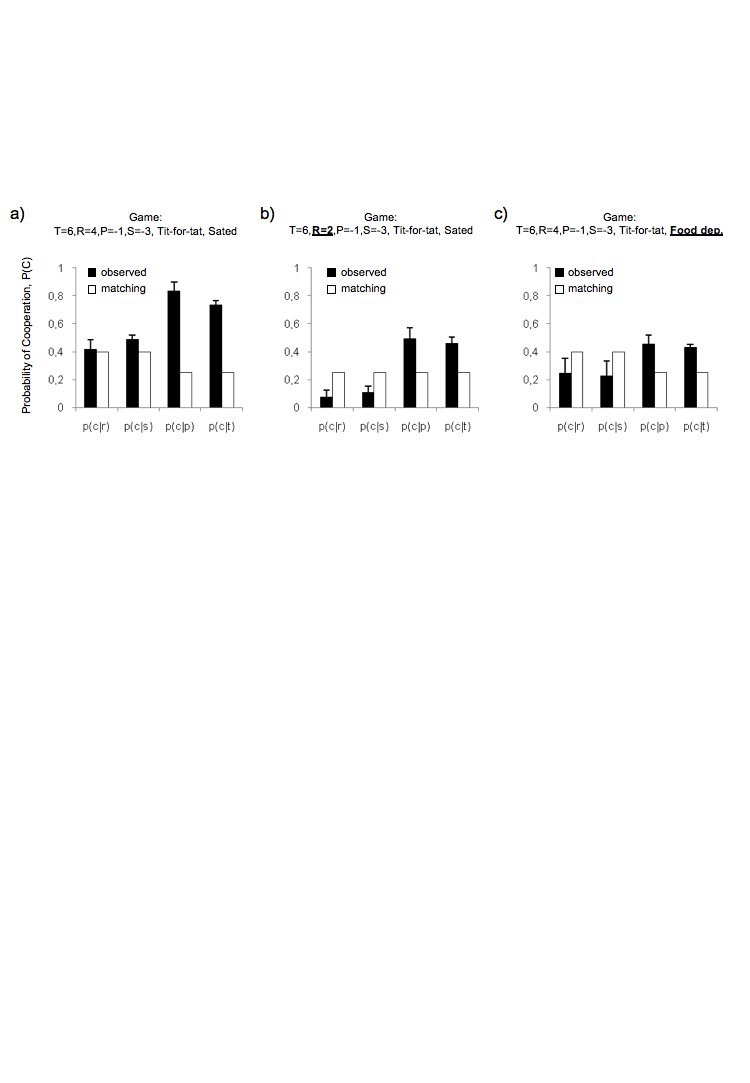

Supplement: Figure S3 — Comparison between observed behaviour and matching behaviour. The figure shows the observed probability of cooperation after each outcome, Reward, Sucker, Punishment and Temptation, black bars (mean±s.e.m), together with the expected probability of cooperation if rats would be matching for reward (p(C0|R-1) and p(C0|S-1)) or punishment (p(C0|P-1) and p(C0|T-1)) magnitudes, white bars. The observed behaviour approached that of matching only for the game in a), when rats were choosing between 6 or 4 food pellets, i.e., after a reward or sucker trial. Note that this analysis is not possible for the game where rats were playing against a pseudo-random stooge, because all transitions between outcomes were possible, and thus, rats had to choose between rewards or punishments of different magnitudes, but also between rewards and punishment (in this case outcomes are not comparable, therefore matching does not apply). (0.08 MB TIF) [file pone.0008483.s003.tif]
